# Brimonidine protects against loss of Thy-1 promoter activation following optic nerve crush

**DOI:** 10.1186/1471-2415-13-26

**Published:** 2013-06-27

**Authors:** Yi Dai, James D Lindsey, Karen X Duong-Polk, Panida Chindasub, Christopher Kai-Shun Leung, Robert N Weinreb

**Affiliations:** 1 Hamilton Glaucoma Center and Department of Ophthalmology, University of California San Diego, La Jolla, CA 92093, USA; 2 Department of Ophthalmology, Eye & ENT Hospital, Fudan University, Shanghai, China; 3 Department of Ophthalmology and Visual Sciences, Chinese University of Hong Kong, Hong Kong, China

**Keywords:** Retina, Brimonidine, Optic nerve crush, Neurodegeneration

## Abstract

**Background:**

The loss of RGCs expressing Thy-1 after optic nerve injury has an initial phase of rapid decline followed by a longer phase with slower reduction rate. This study used longitudinal retinal imaging of mice expressing cyan fluorescent protein under control of the Thy-1 promoter (Thy1-CFP mice) to determine how the α2-adrenergic agonist brimonidine influences loss of Thy1 promoter activation.

**Methods:**

Baseline images of the fluorescent retinal neurons in 30 Thy1-CFP mice were obtained using a modified confocal scanning laser ophthalmoscope. Next, brimonidine (100 ug/kg, IP) was administered either one time immediately after optic nerve crush, or immediately after optic nerve crush and then every 2 days for four weeks. A control group received a single saline injection immediately after optic nerve crush. All animals were imaged weekly for four weeks after optic nerve crush. Loss of fluorescent retinal neurons within specific retinal areas was determined by counting.

**Results:**

At one week after optic nerve crush, the proportion of fluorescent retinal neurons retaining fluorescence was 44±7% of baseline in control mice, 51±6% after one brimonidine treatment, and 55±6% after brimonidine treatment every other day (*P*<0.05 for both brimonidine treatment groups compared to the control group). Subsequently, the number of fluorescent retinal neurons in the group that received one treatment differed insignificantly from the control group. In contrast, the number of fluorescent retinal neurons in the group that received repeated brimonidine treatments was greater than the control group by 28% at two weeks after crush and by 32% at three weeks after crush (*P*<0.05 at both time points). Rate analysis showed that brimonidine slowed the initial rate of fluorescent cell decline in the animals that received multiple treatments (*P*<0.05). Differences in the rate of loss among the treatment groups were insignificant after the second week.

**Conclusion:**

Repeated brimonidine treatments protect against loss of fluorescence within fluorescent retinal neurons of Thy1-CFP mice after optic nerve crush. As most of fluorescent retinal neurons in this system are RGCs, these findings indicate that repeated brimonidine treatments may protect RGC health following optic nerve crush.

## Background

Loss of retinal ganglion cells (RGCs) is a well-known consequence of optic nerve damage that occurs in a number of diseases including glaucoma, ischemic optic neuropathy and compressive optic neuropathy [1-3]. A goal for neuroprotective treatment of these diseases is to protect RGCs from damage following optic nerve injury. Brimonidine, an α2-adrenergic receptor agonist, and widely used ocular hypotensive agent, has been reported to protect against RGC death in rodent models of optic nerve crush, acute retinal ischemia and glaucoma [4-8]. However, whether brimonidine can also protect RGCs from early degenerative events remains poorly understood.

Thy-1 is a cell surface glycoprotein that in retina is expressed on the cell bodies of healthy adult RGCs [9,10]. During development, Thy-1 mRNA and protein expression in RGCs increases to adult expression at two weeks after birth in coincidence with synapse formation at RGC innervation targets within the brain [11,12]. Optic nerve crush triggers a decline in Thy-1 mRNA within the first day after optic nerve crush that is followed a few days later by decline in Thy-1 protein, and one to two weeks later by RGC death [13]. Thus, the transcription of Thy-1 mRNA and expression of Thy-1 protein by adult RGCs are early-responding markers of adult RGC health. Moreover, interventions that diminish or delay loss of Thy-1 promoter activation after optic nerve crush may reflect protection of RGC health.

Longitudinal assessment of Thy-1 promoter activation in the retina has become more practical with the recent development of a method to noninvasively image fluorescent retinal neurons in transgenic mice expressing cyan fluorescent protein (CFP) under the control of the Thy-1 promoter (Thy1-CFP mice) [14]. These images show bright spots corresponding to individual fluorescent neurons as well as fluorescent retinal nerve fiber layer axon bundles converging at the optic nerve head. Following optic nerve crush, a major portion of these fluorescent retinal neurons cease expression of CFP over the first one to two weeks with more gradual loss occurring over the subsequent two to three weeks [14]. An important advantage of this approach for longitudinal evaluation of the same retinal neurons is that it avoids the uncertainty associated with having to compensate for the normal large variation in RGC numbers among individual mice by using large experimental group sizes for each time point.

The present study has used this longitudinal in vivo imaging approach to determine whether single or multiple brimonidine treatments diminish or delay the time-dependent loss of fluorescent retinal neurons in Thy1-CFP mice following optic nerve crush. The optic nerve crush model was used because the simultaneous injury to all optic nerve axons facilitates detection of changes in the time-dependent responses to optic nerve crush.

## Methods

### Animals

This study used hemizygous B6.Cg-Tg (Thy1-CFP)23Jrs mice (both male and female) approximately 3 months old. These animals were bred at UCSD from the same stocks that provided animals for prior studies [14,15]. All experimental procedures conformed to the ARVO Statement for the Use of Animals in Ophthalmic and Vision Research and were approved by the UCSD Institutional Animal Use and Care Committee. The animals were housed using standard vivarium enclosures, provided standard food and water ad libitum, and maintained with 12 hours light and 12 hours dark daily light cycle. Animal health was checked daily by trained personnel under the supervision of a laboratory research veterinarian.

### Treatments

Three groups of Thy1-CFP23Jrs mice were studied following optic nerve crush. In the first group, brimonidine (100 μg/kg) was administered by intraperitoneal injection only immediately after optic nerve crush. In second group, brimonidine (100 μg/kg) was administered immediately after optic nerve crush and then every 2 days for the remainder of the study (QOD). The control group mice received one saline injection immediately after optic nerve crush.

### BCSLO imaging

Imaging by bCSLO was performed as previously described [14,15]. Animals were gently restrained by hand and no anesthesia was used. A single scan corresponding to a retinal area of about 2 mm^2^ was obtained in less than 5 seconds. The short exposure period needed for focusing and image collection was generally well tolerated by the mice. Both eyes were imaged at each imaging session. This allowed monitoring of any fluorescent cell loss for reasons unrelated to the optic nerve crush.

### Experimental design

Ocular biomicroscopic examination was performed to exclude any animals with abnormal ocular appearance. Power analysis indicated 80% power to detect a 30% difference in the presence of a standard deviation of 8% with *P <* 0.05 would be achieved if the sample size was 5 mice/group or greater. A prior study using this experimental system indicated there would likely be some losses due to technical issues [14]. Hence, 30 animals with normal ocular appearance were randomly assigned from each of the litters contributing to this population to each of the three treatment groups until there were 10 animals/group. Average body weight for all animals was 19.6 ± 2.9 grams and the means for each of the groups differed from this value by less than 3%. Following retinal imaging by bCSLO, monocular intraorbital optic nerve crush was performed as previously described [15]. Care was taken to perform the crush sufficiently distal to the globe so that retinal circulation was preserved. The retinas of the eyes that received optic nerve crush were imaged by bCSLO at 1, 2, 3 and 4 weeks after crush. Body weight of each included animal was measured at the time of baseline image collection and then measured weekly for the remainder of the study as a measure of animal health. After initiation of the study, 1 animal in the control group, 4 animals in the single treatment group, and 3 animals in the repeated treatment group were excluded due to the development of cataract or corneal opacity during the course of the study. Thus, there remained 9 animals in the control group, 6 animals in the single treatment group, and 7 animals in the repeated treatment group.

### Analysis

All of the images for each eye that received optic nerve crush were reviewed at the end of the experiment to identify retinal regions in which there was good quality imaging of the fluorescent cells from each of the examination sessions. Selected region images meeting this criterion and containing at least 120 fluorescent cells at baseline were identified for each eye based on retinal features including blood vessels, fluorescent axon bundles, and the few cells that retained their fluorescence throughout the study. Using Photoshop (CS4, Adobe, San Jose, CA), these defined areas were cut from each image and the images were inverted to facilitate counting. Masked counts of fluorescent cells in these defined retinal areas were made by a counter unaware of the experimental condition or time point of the images. After unmasking, the counts at each time point for each eye were expressed as percent of the baseline count in that same eye.

### Statistical evaluation

The data are expressed as mean ± standard deviations (SD). Body weight was compared at each time point using the Students t test. The fluorescent retinal neuron counts at each time point in the group that received a single briminidine treatment and in the group that received repeated brimonidine treatments were compared against the corresponding counts in the vehicle control group by one-way analysis of variance (ANOVA) and the Student-Newman-Keuls test (SNK, Primer of Biostatistics 3.01). *P* < 0.05 was considered to be statistically significant.

## Results

### Body weight

**Figure 1:**
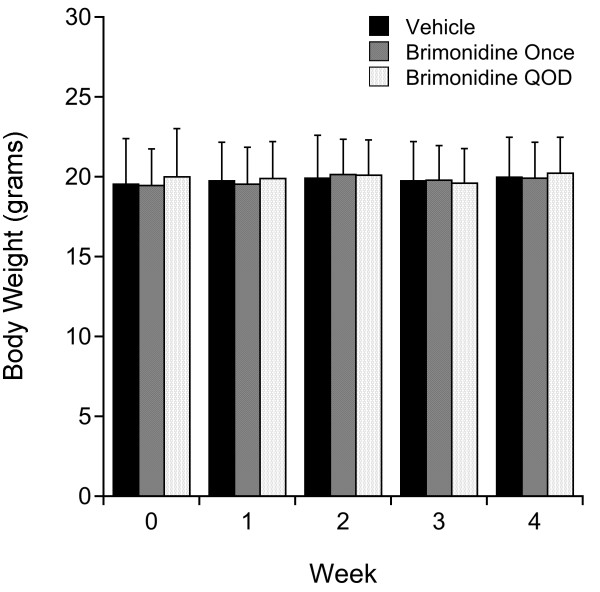
**Absence of body weight change during the course of the study or difference among the treatment groups (*P*** **> 0.93 for each time point, ANOVA).**

The body weight of animals receiving either vehicle treatment (n = 9), single brimonidine treatment (n = 6), or repeated brimonidine treatment (n = 7) did not change significantly during the course of the study (*P* = 0.93 to 0.97 for each time point, t test, Figure 1).

### Images

**Figure 2:**
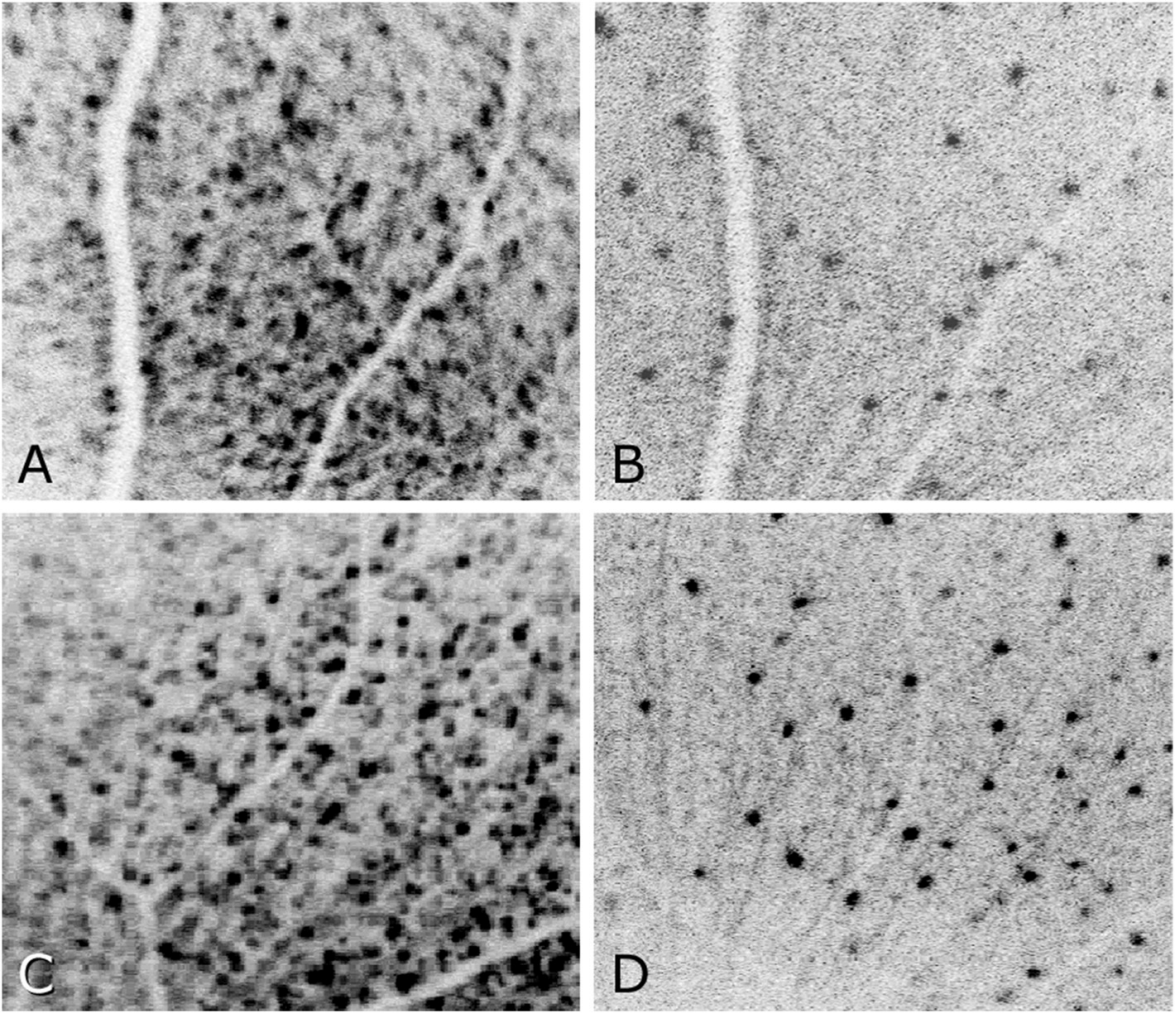
**Inverted fluorescent retinal neurons imaged in vivo using bCSLO at baseline (A and C) and in the same eyes at 4 weeks (B and D) after optic nerve crush from a mouse that received saline treatment (A, B) and a mouse that received repeated brimonidine treatment (C,D).** Fluorescent neurons appear as black spots, axon bundles appear as dark thin lines radiating from the optic nerve head (seen more easily in **B** and **D**), while retinal blood vessels appear as bright thick lines branching from the optic nerve head.

The bCSLO images contained bright fluorescent spots corresponding to individual fluorescent retinal neurons and were inverted to facilitate counting (Figure 2). Each fluorescent retinal neuron appeared in the inverted images as dark spots while nerve fiber layer axon bundles appeared as dark lines radiating from the optic nerve head. Retinal blood vessels blocked background fluorescence (that likely originated from fluorescent RGC dendrites in the inner plexiform layer [14]) and hence appeared as bright branching lines in the inverted images. The structural features of the axon bundles and blood vessels allowed the reproducible identification of retinal areas that contained at least 120 fluorescent retinal neurons in the baseline images and that were in focus in the images from subsequent time points for each eye.

### Counts

**Figure 3:**
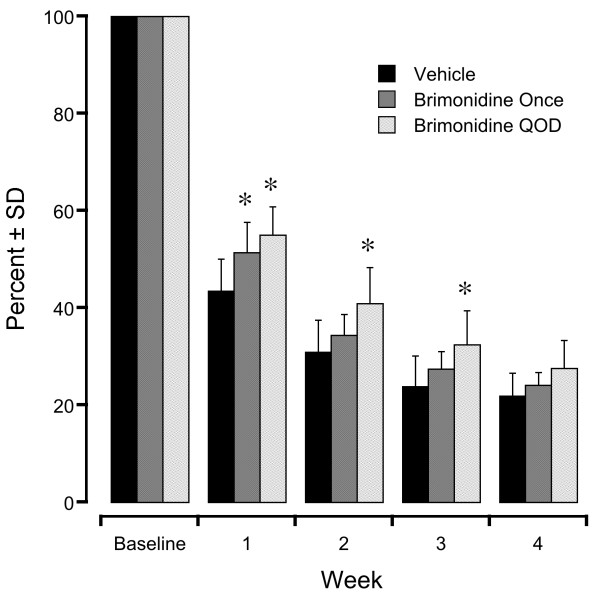
**Preservation of fluorescent retinal neurons in defined retinal areas of mice following optic nerve crush with a single brimonidine treatment or with brimonidine treatment every other day (QOD).** Asterisks indicate *P <* 0.05 compared with the control group.

Fluorescent retinal neuron counts in the defined retinal areas in the vehicle control eyes at 1, 2, 3, and 4 weeks after optic nerve crush were 44 ± 7%, 31 ± 7%, 24 ± 7%, and 22 ± 5% of baseline counts (n = 9, black bars, Figure 3). In the mice that received a single brimonidine treatment, fluorescent retinal neuron counts at the same time points were 51 ± 6%, 34 ± 4%, 27 ± 4%, and 24 ± 3% of baseline counts (n = 6, dark gray bars, Figure 3). These counts were 18% greater than the vehicle control eye counts at week 1 (*P* < 0.05, SNK test at each time point) but were not significantly different at weeks 2, 3, and 4 following optic nerve crush. In the mice that received repeated brimonidine treatments, fluorescent retinal neuron counts at the same time points were 55 ± 6%, 44 ± 7%, 32 ± 7%, and 28 ± 6% of baseline counts (n = 7, light gray bars, Figure 3). These counts were 27%, 28%, and 32% greater than the vehicle control counts at week 1, week 2, and week 3 following optic nerve crush (*P <* 0.05 at each time point, SNK test).

### Rate analysis

**Figure 4:**
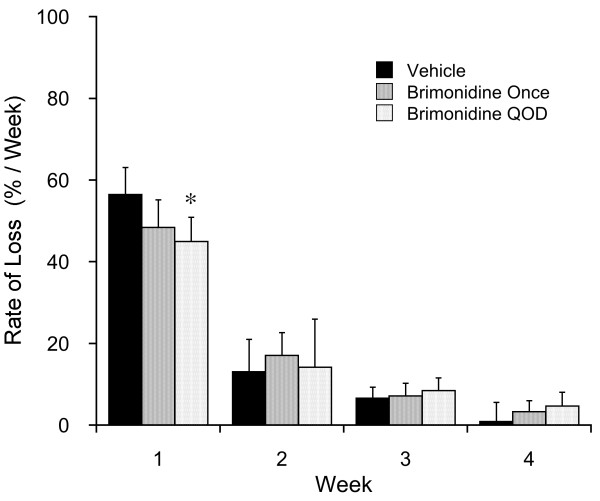
**Rate analysis expressed as percent of baseline fluorescent retinal neuron loss per week.** Brimonidine QOD induced significantly slower fluorescent retinal neuron loss than either vehicle or a single brimonidine treatment (asterisk indicates *P <* 0.05).

Longitudinal evaluation of changes in the rate of fluorescent retinal neuron loss in the vehicle treated mice declined from 57 ± 7 percent of baseline counts per week in the first week, to 13 ± 8 percent per week in the second week, 7 ± 3 percent per week in the third week, and 1 ± 5 percent per week in the fourth week (Figure 4). This result is consistent with prior reports showing faster loss during the first one to two weeks and much slower loss rates during later weeks [15,16]. Repeated brimondine treatment slowed the rate of loss during the first week from 57 ± 7%/week to 45 ± 6%/week (*P* < 0.05, SNK test). During the second, third, and fourth weeks, the rate of fluorescent retinal neuron loss did not significantly differ among the treatment groups.

## Discussion

These results show that brimonidine treatment protects against the loss of Thy-1 promoter activation that occurs following optic nerve crush. A single treatment provided significant protection at one week after crush, but this effect became insignificant at later time points. Repeated brimonidine treatment, however, provided protection that was both larger and longer lasting. Specifically, the ~30% protection observed at one week was maintained for the next two weeks. Rate analysis confirmed that brimonidine significant slowed the rate of loss of cells with Thy-1 promoter activation during the first week after crush and became the same as the rate of loss in the control group at 2 and 3 weeks after crush.

Immunohistochemical studies indicate that 20% of CFP-expressing cells in healthy Thy1-CFP23Jrs mice are cholinergic amacrine cells [17]. These cholinergic amacrine cells appear to be refractory to cell loss in a Thy1-CFP DBA/2J mouse line in which there is spontaneous loss of RGCs [18]. Hence, it seems reasonable to estimate the loss RGCs expressing CFP in the current study by subtracting 20% of the baseline counts from the current observations. Performing this calculation projects that RGCs expressing CFP in the vehicle treated mice were 24%, 11%, 4%, and 2% of baseline at 1, 2, 3, and 4 weeks after crush. This result is consistent with prior studies of Thy-1 expression after optic nerve crush [13,16]. Applying this 20% adjustment to the results for the repeated brimonidine treatment group projects that RGCs expressing CFP were 35%, 21%, 12%, and 8% of baseline at 1, 2, 3, and 4 weeks after crush. Thus, the present results indicate that systemic brimonidine treatment can provide substantial protection against loss of Thy-1 promoter activation following optic nerve crush.

In agreement with an earlier study [15], the rate of loss of fluorescent neurons following optic nerve crush was biphasic with more rapid loss initially followed by a period of slower loss. These two rates may reflect progression in the degenerative process or differences in the rates of primary and secondary degeneration [3,19]. The present observation that brimonidine significantly delayed the earlier more rapid phase but did not significantly change the rate of loss in the later slower phase suggests that its protective effect may be more important during the earlier phase. Nevertheless, the increased preservation of CFP-expressing RGCs obtained in the first week was largely maintained over the next two weeks in the animals that received continued brimonidine treatments. Thus, these results indicate that brimonidine treatment may protect RGC health both during the first few days after crush as well as during subsequent weeks.

The mechanism underlying the neuroprotective effect of brimonidine remains unclear. Prior studies suggest direct action of brimonidine in the protection of injured neurons since protection against RGC death can be blocked by α2-antagonists [5,6]. The lack of a general metabolic effect is supported by the stability of body weight during the course of the present study. Brimonidine can increase the expression of growth factors such as brain-derived neurotrophic factor and fibroblast growth factor [20-22]. Brimonidine may also presynaptically inhibit glutamate release [7,23], as well as modulate postsynaptic NMDA receptor function [24]. In view of the protection of both early and later degeneration phases delineated in the present study, it will be of interest to determine how quickly these physiological responses are altered after the initiation or cessation of brimonidine treatment.

Several limitations of the models employed should be considered when interpreting the present results. First, the optic nerve crush model simultaneously triggers RGC death that occurs within several weeks. This differs from the typical prolonged and asynchronous course of primary open angle glaucoma (POAG) where less than 0.02% of RGCs may be undergoing apoptosis at any one time [3,25]. Another consideration is that loss of CFP expression in the Thy1-CFP mouse model employed in the present study may differ from the loss of RGC function [26]. Finally, the present experimental system does not address possible changes in the rate of brain alterations that may occur following either optic nerve crush or glaucoma [27-29]. Nevertheless, because Thy-1 depletion following optic nerve crush significantly precedes the onset of RGC death, the present results suggest that systemic brimonidine treatment may delay the loss of RGC health immediately following optic nerve crush.

## Conclusions

This study has demonstrated that repeated brimonidine treatments protect against loss of fluorescence among fluorescent retinal neurons in Thy1-CFP mice after optic nerve crush. As most of fluorescent retinal neurons in this system are RGCs, these findings indicate that repeated brimonidine treatments may protect RGC health following optic nerve crush. Moreover, this effect is greatest during the first week following optic nerve crush and persists during subsequent weeks. These results support further studies to investigate whether brimonidine can be exploited to protect RGC health following other types of optic nerve injury or to facilitate RGC recovery in clinical conditions such as compressive optic neuropathy or glaucoma.

## Competing interests

This work was supported by a grant to JDL from Allergan. The authors do not have a patent interest in this research. The authors do not have other financial competing interests regarding the work presented in this manuscript.

## Authors’ contributions

YD and JL designed the study, analyzed the data, and wrote the manuscript. YD, KD-P, and PC contributed to development of the methods and conducted the study including surgery, imaging, and image analysis. CL and RW contributed to the analysis of the data and the preparation of the manuscript. All authors read and approved the final manuscript.

## Pre-publication history

The pre-publication history for this paper can be accessed here:

http://www.biomedcentral.com/1471-2415/13/26/prepub
